# Transcriptomic Analysis of Tambaqui (*Colossoma macropomum*) Exposed to Trichlorfon-Induced Toxicity

**DOI:** 10.3390/ani15121807

**Published:** 2025-06-19

**Authors:** Hallana Cristina Menezes da Silva, Igor Kelvyn Cavalcante Lobo, André Gentil da Silva, Ana Lúcia Silva Gomes, Wallice Paxiúba Duncan, Juliana Costa Silva, Fabrício M. Lopes, Roberto Ferreira Artoni, Daniele Aparecida Matoso

**Affiliations:** 1 Programa de Pós-Graduação em Genética, Conservação e Biologia Evolutiva, Instituto Nacional de Pesquisas da Amazônia, Manaus 69067-375, AM, Brazil; 2 Programa de Pós-Graduação em Genética, Laboratório de Algoritmos em Biologia, Universidade Federal de Minas Gerais, Belo-Horizonte 31270-800, MG, Brazil; lobo.ikc@gmail.com; 3 Laboratório de Parasitologia de Animais Aquáticos, Universidade Federal do Amazonas, Manaus 69067-005, AM, Brazil; asswgentil@gmail.com; 4 Laboratório de Parasitologia de Animais Aquáticos, Departamento de Parasitologia, Universidade Federal do Amazonas, Manaus 69067-005, AM, Brazil; anapaimagomes@gmail.com; 5 Laboratório de Morfologia Funcional, Departamento de Morfologia, Universidade Federal do Amazonas, Manaus 69067-005, AM, Brazil; wduncan@ufam.edu.br; 6 Departamento de Informática, Universidade Federal do Paraná, Curitiba 81531-990, PR, Brazil; julianacostasilvati@gmail.com; 7 Programa de Pós-Graduação em Bioinformática, Departamento Acadêmico de Computação, Universidade Tecnológica Federal do Paraná, Cornélio Procópio 86300-000, PR, Brazil; fabricio@utfpr.edu.br; 8 Laboratório de Genética e Evolução, Departamento de Biologia Estrutural, Molecular e Genética, Universidade Estadual de Ponta Grossa, Ponta Grossa 84010-330, PR, Brazil; rfartoni@gmail.com; 9 Laboratório de Biotecnologia e Citogenômica Animal, Departamento de Genética, Universidade Federal do Amazonas, Manaus 69067-005, AM, Brazil; danielematoso@yahoo.com.br

**Keywords:** toxicology, trichlorfon, fresh water, *C. macropomum*

## Abstract

Trichlorfon is an antiparasitic agent widely used to control pests and parasites in farmed fish. Tambaqui (*C. macropomum*) is the most commercially important characid species farmed in the Amazon region. Trichlorfon exposure is known to cause physiological damage in fish due to its organophosphate nature. In this study, we used RNA-Seq to investigate the hepatic response of tambaqui following exposure to 0.435 mg/L of trichlorfon. The analysis revealed activation of several metabolic pathways, particularly those related to tumor processes, immune responses, and apoptosis. Additionally, we identified upregulation of solute carrier (SLC) genes, which may facilitate trichlorfon entry into hepatocytes. These findings enhance our understanding of fish responses to antiparasitic agents and support further research into the molecular impacts of organophosphate compounds in aquaculture species.

## Simple Summary

Trichlorfon is widely used in aquaculture for parasite control, yet its physiological impacts on fish remain incompletely understood. In this study, we assessed the hepatic transcriptomic response of tambaqui (*Colossoma macropomum*) following trichlorfon exposure. RNA-Seq analysis revealed upregulation of genes involved in inflammation, immune response, apoptosis, and xenobiotic metabolism. Notably, certain transporter proteins may mediate trichlorfon uptake into hepatocytes, triggering downstream cellular effects. We also detected activation of genes associated with genotoxic stress, suggesting potential DNA damage. These findings offer novel insights into the molecular toxicity of trichlorfon and highlight the need for safer aquaculture practices to ensure fish health and food safety.

## 1. Introduction

Pesticides are chemical substances used globally, both locally and on a large scale, to control pests. More than 4 million tons of pesticides are produced annually, with the majority being used by China, the United States, and Brazil [1]. Organophosphate compounds (OPs) are frequently employed in agriculture and aquaculture to control parasites. Most of these compounds are considered highly toxic and have been reported to bioaccumulate in the environment, including in sediments and aquatic ecosystems [1,2].

One of the most widely used organophosphate insecticides, both in domestic settings and industrial applications, is trichlorfon (2,2,2-trichloro-1-dimethoxyphosphoryl ethanol). It is applied in agriculture to control pests associated with production systems [3] and is also extensively used in aquaculture to control aquatic insects, Odonata nymphs, flatworms, leeches, and parasites [3,4]. In Brazil, the use of this organophosphate is authorized under the “Agrochemical Law” (Law No. 7802/1989) and its regulatory decree (Decree No. 4074/2002), which governs the implementation of the law. The agencies responsible for its regulation are ANVISA (Brazilian Health Regulatory Agency) and IBAMA (Brazilian Institute for the Environment and Renewable Natural Resources). However, there is currently no maximum residue limit (MRL) established by law specifically for the use of trichlorfon in fish.

Acetylcholinesterase (AChE) is the primary enzymatic target of trichlorfon, whose inhibition leads to continuous nerve impulse transmission [5]. As a result of these physiological disruptions and enhanced neural signaling [6], trichlorfon can induce acute effects in aquatic species, particularly fish. These effects include the loss of equilibrium during swimming and excessive muscle contraction [7].

Despite its documented efficacy against fish parasites, several studies have reported indirect contamination effects in fish treated with trichlorfon. In *Cyprinus carpio* L., elevated expression levels of *hsp70* and *cytochrome p450*, along with damage to erythropoietic tissue, have been observed [8]. In zebrafish (*Danio rerio*), congenital malformations and delayed embryonic development have been reported [9]. Neurotoxic effects, including oxidative brain damage and neurotransmitter disruption, have been documented in jundiá (*Rhamdia quelen*) [10]. According to Silva et al. (2020) [7], exposure of tambaqui (*C. macropomum*) to trichlorfon caused loss of swimming balance and organ damage, correlating with AChE inhibition in the brain and muscle tissues of this species [11]. Furthermore, multiple studies have demonstrated that trichlorfon exposure may induce hepatic damage in various fish species [12,13,14,15,16,17,18].

Tambaqui is a neotropical fish species extensively farmed worldwide. Currently, the majority of tambaqui production occurs in China, surpassing that of South America [19]. According to the Brazilian Institute of Geography and Statistics (IBGE), in 2021, tambaqui was the most commonly farmed native fish species in Brazil, with a reported production of 6880 tons. It serves as a major protein source throughout the Amazon region, where it is often subjected to parasite control in local aquaculture systems with limited sanitary oversight [3].

Given these circumstances, understanding the genetic mechanisms involved in the neurotoxic effects of trichlorfon is of critical importance. Therefore, the present study aimed to identify the key metabolic pathways and genes associated with trichlorfon-induced responses under controlled experimental conditions.

## 2. Materials and Methods

### 2.1. Ethics Statement

All procedures were conducted in strict accordance with ethical standards approved by the Animal Ethics Committee of the Federal University of Amazonas, Manaus, Brazil, under protocol number 030/2018.

### 2.2. Experimental Design and Exposure to Trichlorfon

To ensure the reliability of the data and eliminate potential bias from prior exposure, it was essential that the fish used in this study originated from a fish farm where trichlorfon was not used for parasite control. *C. macropomum* (tambaqui) specimens were obtained from the Experimental Farm of the Federal University of Amazonas, located at BR-174 Highway, km 38, Presidente Figueiredo, Manaus-AM, Brazil. The fish were collected from naturally cultivated ponds and transported to the Humid Laboratory of Parasitology, Morphology, and Fish Genetics at the Federal University of Amazonas in Manaus. Upon arrival, the specimens were acclimated for 60 days in 310 L open polyethylene tanks with continuous water and air circulation. They were fed a commercial diet containing 36% crude protein formulated to promote growth. After the acclimation period, the fish were randomly distributed into two separate polyethylene tanks to initiate the experimental procedures.

**Figure 1:**
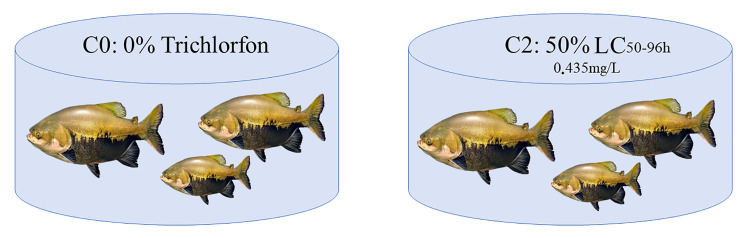
Sample distribution for transcriptome analysis. C0: control condition; C2: 50% of LC_50–96h_ concentration (0.435 mg/L) of trichlorfon.

The trichlorfon exposure level was established at 50% of the LC_50–96h_ value (0.870 mg/L), as previously reported by Silva et al. (2020) [7]. Accordingly, fish were exposed to a nominal concentration of 0.435 mg/L of trichlorfon for 96 h. The trichlorfon solution was prepared in advance and added at the start of the experiment, following the suspension of water circulation and adjustment of the final volume to 60 L per tank, with three fish housed per tank. Fish were randomly assigned to two groups: an experimental group (0.435 mg/L trichlorfon) and a control group (no trichlorfon added) (Figure 1). Three fish from each group were euthanized by spinal transection. The mean weight of the specimens was 222.4 ± 0.08 g, with a standard length of 19.47 ± 0.03 cm and a total length of 23.06 ± 0.08 cm. Liver samples were collected from each individual for subsequent analysis. Throughout the experiment, water quality parameters were monitored using a multiparameter probe (PH/ORP, OD, CE, GPS—HI9829-10041—Hanna Instruments, Woonsocket, RI, USA), including temperature (°C), pH, and dissolved CO_2_.

### 2.3. Sample Collection, RNA Extraction and cDNA Construction

Liver samples were homogenized in Trizol Reagent^®^ (Invitrogen by Applied Biosystems, Woburn, MA, USA) for total RNA extraction, following the manufacturer’s protocol. RNA integrity was then assessed by electrophoresis on a 1% denaturing agarose gel stained with SYBR^®^ Safe Gel Stain (Invitrogen by Applied Biosystems, Eugene, OR, USA). The samples were subsequently sent to the Central Laboratory of High-Performance Technologies and Life Sciences (LaCTAD) at the University of Campinas (UNICAMP), in Campinas, São Paulo, Brazil, for transcriptome sequencing. RNA quality was evaluated using a BioAnalyzer 2100 (Agilent Technologies, Santa Clara, CA, USA) to confirm integrity and determine the RNA Integrity Number (RIN), with values above 7 considered suitable for sequencing. Additionally, RNA quantity was measured using a Qubit^®^ fluorometer (Invitrogen by Applied Biosystems, Woburn, MA, USA) to ensure the minimum concentration and mass required for library preparation.

### 2.4. Library Construction and Sequencing

Library preparation was performed according to the manufacturer’s protocol using the MS-102-3003 MiSeq Reagent Kit v3 (600-cycle) from Illumina. Following preparation, libraries were analyzed using the BioAnalyzer 2100 (Agilent Technologies, Santa Clara, CA, USA) and quantified with a Qubit fluorometer (Invitrogen by Applied Biosystems, Woburn, MA, USA) and quantitative PCR using the KAPA Fast Universal Kit (Sigma-Aldrich, Saint Louis, MO, USA). After confirming the quality and efficiency of the library preparation, sequencing was conducted on the Illumina MiSeq platform using paired-end reads (2 × 300 bp), generating approximately 7 to 8 million reads per sample.

### 2.5. Transcriptome Data Analysis and Identification of Differentially Expressed Genes (DEGs)

FastQC was used to assess the quality of the raw sequencing reads. Read trimming and quality filtering were performed using Trimmomatic. Transcript quantification was carried out with Salmon (v1.10.1), using the *C. macropomum* reference genome available in the NCBI database (BioProject number PRJEB40318; assembly accession GCA_904425465.1).

Differential gene expression analysis was performed using DESeq2 in the R environment (v1.40.2) [20]. The results included a volcano plot displaying all differentially expressed genes (DEGs) and a heatmap highlighting selected genes of interest. Functional enrichment analyses were conducted using Gene Ontology (GO) and the Kyoto Encyclopedia of Genes and Genomes (KEGG) to identify enriched biological pathways associated with the DEGs.

### 2.6. Statistical Analysis

Principal component analysis (PCA) was used to validate the distribution and grouping of biological replicates in the transcriptome dataset.

## 3. Results

### 3.1. Transcriptomie Analysis

**Figure 2:**
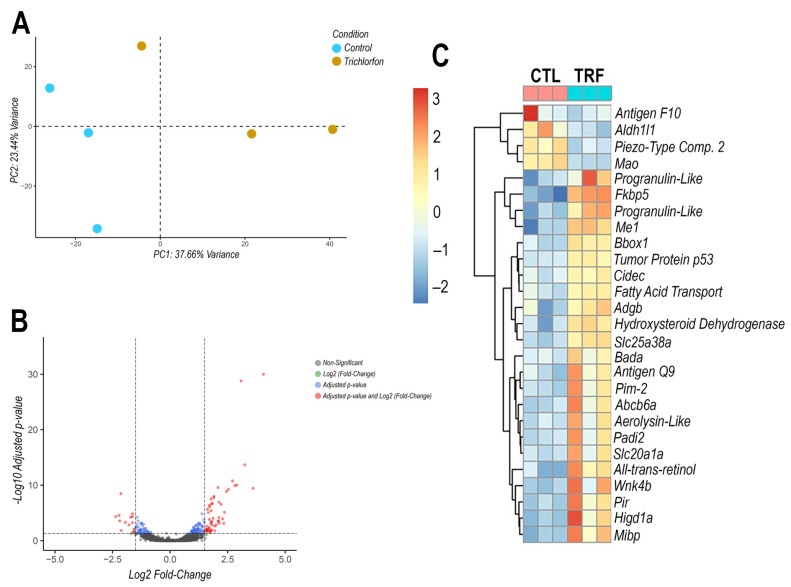
Principal component analysis (**A**), Volcano plot of the differentially expressed genes (DEGs) analyzed (**B**), and heatmap of the selected DEGs (**C**). (**A**) Principal component analysis (PCA) of the experimental (yellow) and control (blue) conditions. (**B**) Volcano plot demonstrating the RNA-Seq-mapped differentially expressed genes (DEGs). The non-significant genes are indicated in gray, while the genes that demonstrated significance in the Log2 Fold Change value are shown in green. The genes that exhibited significance in the *p*-values are represented in blue, and the genes that demonstrated significance in both the *p*-values and the Log2 Fold Change values are indicated in red. (**C**) A heatmap of the genes most affected by Trichlorfon exposure in terms of differential expression. The untreated control samples are represented by F01, F02, and F03, which are displayed in pink. The samples exposed to Trichlorfon (50% of LC_50–96h_—0.435 mg/L) are represented by F13, F14, and F15, which are displayed in blue.

Transcriptome sequencing of tambaqui liver cells from control samples (not exposed to Trichlorfon) and from samples exposed to 50% of LC_50–96h_ (0.435 mg/L) for 96 h revealed a significant number of genes with differential expression in response to the exposure. A principal component analysis (PCA) was conducted to confirm the distribution of the sequenced samples prior to the analysis of differential expressed genes (DEGs; Figure 2A). The generated graph revealed a distinction between the control and experimental groups, suggesting divergences in the mapped DEGs.

A total of 176 differentially expressed genes (DEGs) were identified in comparison to the control group (Supplementary Material Table S1). Among these, 116 genes were upregulated and 60 were downregulated (*p*-value < 0.05). Figure 2B presents a volcano plot illustrating all DEGs detected in the experimental group, providing a comprehensive overview of the transcriptional response to trichlorfon exposure. The analysis revealed genes whose expression levels were significantly altered, suggesting their involvement in various metabolic pathways.

The genes showing the highest levels of differential expression—both upregulated and downregulated—were selected from the set of mapped differentially expressed genes (DEGs). Figure 2C presents a heatmap displaying the expression profiles of the 27 selected genes. Table 1 lists the gene names along with their corresponding log₂ fold change values.

### 3.2. Gene Onthology and KEGG Analysis

**Figure 3:**
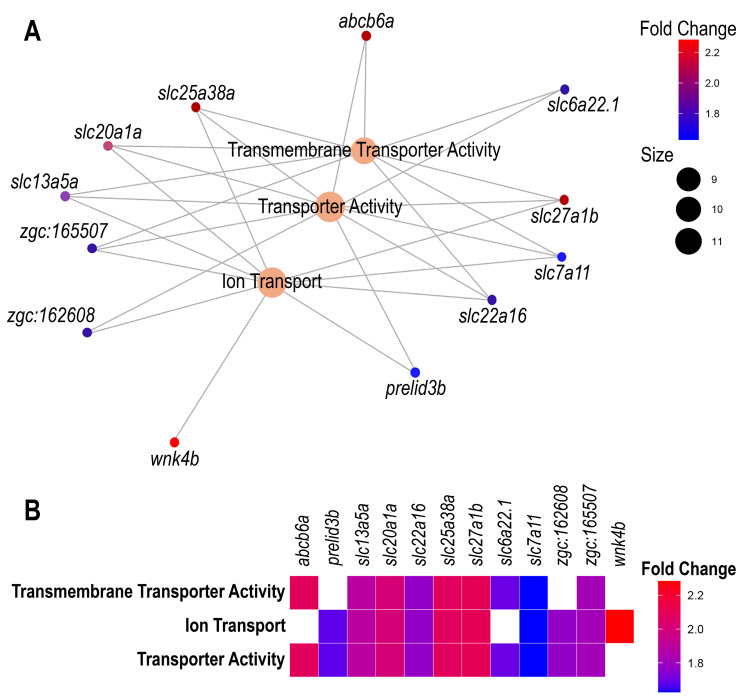
Gene Ontology graph (GO). (**A**) Map of enriched functions and associated genes in tambaqui liver cells exposed to Trichlorfon. (**B**) A scheme of the enriched functions and genes associated with their corresponding Log_2_ Fold Change values. Genes: *abcb6a*—ATP-binding ATP cassete, sub Family B (MDR/TAP), member 6a; *slc6a22.1*—solute-carrier Family 6 member 22, tandem duplicate 1; *slc27a1b*—solute-carrier family 27 member 1b; *slc7a11*—solute-carrier family 7 member 11; *slc22a16*—solute-carrier family 22 member 16; *prelid3b*—PRELI domain containing 3B; *wnk4b*—WNK lysine deficient protein kinase 4b; *slc13a5a*—solute-carrier family 13 member 5a; *slc20a1a*—solute-carrier family 20 member 1a; *slc25a38a*—solute-carrier family 25 member 38a. The genes *zgc:162608* e *zgc:165507* were uncharacterized.

Gene Ontology (GO) mapping was performed to identify enriched functional categories. Three biological functions were significantly enriched in the liver cells of fish exposed to trichlorfon (*p*-value < 0.05): ion transport, transmembrane transporter activity, and general transporter activity (Figure 3). Most of these functions included genes encoding solute carrier proteins from various families and subtypes, suggesting that these transporters may facilitate the cellular uptake of the trichlorfon molecule.

Specifically, nine upregulated genes were associated with the transmembrane transporter activity pathway, while eleven additional upregulated genes were linked to the broader transporter activity category. Ten upregulated genes were also identified within the ion transport pathway. Figure 3 presents the GO enrichment results, illustrating the log₂ fold change values of the genes associated with each enriched pathway.

Pathway mapping was performed using the Kyoto Encyclopedia of Genes and Genomes (KEGG) database to identify enriched metabolic pathways. A total of 63 enriched pathways were identified in response to trichlorfon exposure. Table 2 lists the enriched pathways along with their corresponding KEGG pathway IDs and the enzymes that showed increased expression. The complete list of enriched pathways is available in Supplementary Material Table S2.

## 4. Discussion

Since the adverse effects of trichlorfon treatment have been recognized, several studies have investigated its indirect impacts on fish and other aquatic organisms. In Brazil, fish species such as *Prochilodus scrofa* (curimbatá), *Piaractus mesopotamicus* (pacu), *Oreochromis niloticus* (Nile tilapia), *Danio rerio* (zebrafish), and *Cyprinus carpio* (common carp) have been exposed to this antiparasitic agent. Trichlorfon has also been administered to *Rhamdia quelen* (jundiá) [10,18,21], *C. macropomum* (tambaqui) [7,11,22,23], *Pseudoplatystoma corruscans* (pintado) [24], and *Arapaima gigas* (pirarucu) [25], with various toxic effects reported. These include alterations in tissue morphology, disruptions in biochemical processes, and changes in gene expression.

In the present study, we provide a comprehensive analysis of trichlorfon-induced effects on the hepatic transcriptome of *C. macropomum*. RNA-Seq analysis revealed differential regulation of genes associated with three major biological processes: xenobiotic transport and metabolism, activation of immune responses, and induction of apoptotic pathways. Notably, genes encoding cytochrome P450 (CYP) enzymes were also differentially expressed, underscoring their key role in the organism’s detoxification response. Together, these findings point to a complex physiological reaction to trichlorfon as a chemical stressor.

The identification of both upregulated and downregulated genes reveals a complex metabolic signature that underscores the multifaceted nature of trichlorfon toxicity. This differential expression pattern suggests a sophisticated cellular response, in which the organism concurrently activates protective pathways while repressing others—potentially as an adaptive strategy to mitigate xenobiotic stress. The simultaneous upregulation of detoxification-related genes and downregulation of certain immune-related genes highlights the need to explore the broader genetic networks and their interconnected regulatory mechanisms. These findings open important avenues for investigating the dynamic interplay between stress response systems. Moreover, the distinct expression profiles of individual genes warrant further investigation to elucidate their specific roles and contributions to the overall toxicological response. Such studies may identify novel biomarkers for environmental monitoring and potential molecular targets for mitigating organophosphate-induced damage in aquatic organisms.

Previous studies have demonstrated that trichlorfon exposure induces significant alterations in both tissue morphology and gene expression, particularly in the liver—a central organ involved in metabolism and detoxification in aquatic organisms—and in the gills, which are key sites for contaminant bioaccumulation [26,27]. Structural or functional impairments in the liver can lead to severe metabolic dysfunctions, including biochemical imbalances and systemic inflammatory responses [26]. Therefore, monitoring hepatic responses to xenobiotic exposure is a critical strategy for elucidating mechanisms of toxicity in fish. The liver’s role in this process is closely linked to the activity of cytochrome P450 enzyme complexes, which are essential for the biotransformation of organophosphates. In *C. macropomum* exposed to Malathion^®^, early activation of Phase II metabolic pathways has been reported, indicating a rapid induction of conjugation mechanisms within the first hours following pesticide exposure [28].

In the present study, the overexpression of solute carrier (SLC) family transporters suggests a potential mechanism for trichlorfon uptake into hepatocytes, with genes such as *slc25a38a*, *slc20a1a*, and *slc22a16* being notably upregulated. This expression pattern reinforces the liver’s central role in xenobiotic metabolism, where trichlorfon is converted into reactive metabolites such as dichlorvos, subsequently leading to oxidative stress in aquatic organisms—a phenomenon also reported by Wang et al. (2022) [29].

In addition, we observed the upregulation of genes associated with immune and inflammatory responses, including *progranulin* and *aerolysin-like* genes. *Progranulin* has been described as a modulator of cytokine signaling and tissue repair processes in teleosts, particularly under hepatic stress induced by environmental contaminants [30]. This expression profile aligns with previous findings of hepatic inflammation and histopathological alterations in fish exposed to organophosphates [31]. These observations are further supported by Silva et al. (2020) [7], who reported organ damage—especially in the liver—characterized by altered coloration and a strong putrid odor in trichlorfon-exposed fish.

Furthermore, the downregulation of the *F10α* gene observed in this study may reflect impaired adaptive immune responses, a pattern consistent with findings in fish exposed to other organophosphates. For example, in *Oreochromis niloticus* exposed to chlorpyrifos, dysregulation of genes involved in immune regulation and inflammatory processes was reported [31]. Such reductions in gene expression may compromise the fish’s ability to mount effective immune responses, underscoring the potential immunotoxic effects of trichlorfon.

Another relevant finding was the upregulation of the proto-oncogenes *tp53* and *pim-2*, as well as the enzyme-coding gene *padi2*. The increased expression of *tp53*, a key gene involved in the response to genotoxic stress, and *pim-2*, which plays a role in cell survival, suggests the activation of cellular defense mechanisms against trichlorfon-induced genotoxicity. In *Cyprinus carpio*, previous studies have reported mitochondrial dysfunction and *p53*-mediated apoptosis following trichlorfon exposure, supporting our results [29]. Additionally, the overexpression of the apoptotic genes *cidec* and *bada* observed in our study indicates progression toward programmed cell death—a common response in fish subjected to high levels of oxidative stress and irreversible DNA damage [32].

Transcriptomic analysis also revealed that *AChE* gene expression in liver tissue was not significantly altered compared to the control group. This finding is consistent with previous studies in *C. macropomum*. Duncan et al. (2020) [11] reported that nominal concentrations corresponding to 30% (0.26 mg/L) and 50% (0.460 mg/L) of the trichlorfon LC_50–96h_ did not affect hepatic AChE enzymatic activity. Similarly, Carvalho et al. (2024) [33] found no change in *AChE* gene expression in *C. macropomum* exposed to trichlorfon. Furthermore, Malathion^®^, another widely used agricultural organophosphate, has also been shown to have no effect on hepatic AChE activity in tambaqui [28]. Based on these findings, we suggest that in the liver—unlike in muscle or brain tissues—*AChE* expression and activity (whether assessed through transcriptomics, gene expression, or in vitro enzymatic assays) may not serve as reliable molecular markers for evaluating organophosphate toxicity. This is likely due to the lower functional dependence on AChE in hepatic tissue, whereas muscle and brain tissues require higher cholinergic activity to maintain contractile and neurophysiological homeostasis, respectively [11,34].

Furthermore, functional enrichment analysis revealed the activation of multiple metabolic pathways in the liver of tambaqui, including glycolysis/gluconeogenesis, fatty acid metabolism, and xenobiotic biotransformation pathways mediated by cytochrome P450 enzymes. These findings indicate a pronounced metabolic reprogramming in response to trichlorfon exposure, corroborating previous studies by Venturini et al. (2014) [35] and Zhang et al. (2023) [27]. The induction of classical hepatic detoxification phases (Phase I and Phase II) underscores the physiological plasticity of tambaqui under chemical stress. However, it also suggests a potential metabolic overload in hepatic tissue, which may compromise the species’ resilience in contaminated environments.

## 5. Conclusions

The exposure of tambaqui to trichlorfon elicited a multifaceted hepatic transcriptomic response, reflecting substantial metabolic reorganization under chemical stress. A total of 176 differentially expressed genes were identified, with particular emphasis on the upregulation of genes involved in xenobiotic uptake—especially members of the SLC transporter family—suggesting an active role of the liver in pesticide internalization. Moreover, the activation of metabolic pathways related to toxic compound biotransformation, including cytochrome P450-mediated detoxification, as well as energy-associated processes such as glycolysis, indicates a coordinated metabolic adaptation to trichlorfon exposure.

Concurrently, the upregulation of genes associated with immune responses (e.g., *progranulin*, *aerolysin-like*) and apoptosis (*tp53*, *pim-2*, *cidec*, *bada*) points to a cellular environment marked by oxidative stress and DNA damage, potentially leading to programmed cell death. The absence of changes in hepatic acetylcholinesterase (*AChE*) expression reinforces the enzyme’s limited utility as a biomarker of liver toxicity, contrasting with its established functional relevance in muscle and neural tissues.

Collectively, these findings demonstrate the significant toxicological impact of trichlorfon on the liver of *C. macropomum*, encompassing immune, metabolic, and cellular dysfunctions. They raise important concerns regarding the repeated use of this antiparasitic agent in Amazonian aquaculture, which may compromise fish health, threaten the sustainability of production systems, and pose risks to food security for human populations that depend on this species as a key protein source.

## Figures and Tables

**Table 1:** Genes with higher up- and down-regulated expression levels from fish liver samples exposed to Trichlorfon.

Gene	Gene Name	Gene ID (NCBI)	Log_2_ Fold Change
Antigen F10	Class I Histocompatibility Antigen, F10 alpha chain-like	LOC118803584	−2.12
Aldh1l1	Aldehyde Dehydrogenase 1 Family, member L1	LOC118808393	−1.94
Piezo-type 2	Piezo-type Mehcanosensitive Ion Channel Component 2	LOC118796923	−2.36
Mao	Monoamine Oxidase	LOC118817391	−2.14
Progranulin-like	Progranulin-like	LOC118817705	2.71
Fkbp5	FKBP Prolyl Isomerase 5	LOC118808338	4.05
Progranulin-like	Progranulin-like	LOC118817703	2.81
Me1	Malic Enzyme 1, NADP(+)-dependent, cytosolic	LOC118796726	3.08
Bbox1	Butyrobetaine (gamma), 2-oxoglutarate dioxygenase 1	LOC118817504	2.21
Tumor protein p53	Tumor protein p53-inducible nuclear protein 2	LOC118822861	1.66
Cidec	Cell Death inducing DFFA like effector c	LOC118808364	1.40
Fatty Acid Transport	Long-Chain Fatty Acid Transport Protein 1-like	LOC118798082	2.10
Adgb	Androglobin	LOC118815891	2.45
Hydroxysteroid Dehydrogenase	Hydroxysteroid Dehydrogenase-like Protein 2	LOC118824306	2.12
Slc25a38a	Solute Carrier Family 25 Member 38a	LOC118805305	2.08
Bada	BLC2 associated agonist of cell death a	LOC118820664	1.75
Antigen Q9	H-2 class I Histocompatibility Antigen, Q9 alpha chain-like	LOC118801148	2.12
Pim-2	Serine/Threonine-protein kinase pim-2-like	LOC118822920	2.37
Abcb6a	ATP biding cassette subfamily B member 6 (LAN blood group) a	LOC118802335	2.08
Aerolysin-like	Aerolysin-like Protein	LOC118799499	2.14
Padi2	Peptidyl Arginine Deiminase, type II	LOC118808437	2.33
Slc20a1a	Solute Carrier Family 20 Member 1a	LOC118813336	2.01
All-trans-retinol	All-trans-retinol 13,14-reductase-like	LOC118820983	2.89
Wnk4b	WNK lysine deficient protein kinase 4b	LOC118806065	2.28
Pir	Pirin	LOC118802703	2.54
Higd1a	HIG1Hypoxia Inducible Domain Family, member 1A	LOC118826377	3.61
Mibp	Muscle-specific beta 1 integrin binding protein	LOC118819161	3.25

The genes that comprise the heatmap and the table were selected from the log2 fold change calculated in the analysis. The genes that exhibited up- and down-regulated expression levels are highlighted.

**Table 2:** Top 37 enriched pathways from KEGG analysis with more than two enzymes involved.

Number	Pathway	Enzyme in Pathway	Pathway ID
1	Glyoxylate and dicarboxylate metabolism	2	map00630
2	Glycolysis/Gluconeogenesis	3	map00010
3	Tryptophan metabolism	2	map00380
4	Lysine degradation	4	map00310
5	Pyruvate metabolism	6	map00620
6	Biotin metabolism	3	map00780
7	Nicotinate and nicotinamide metabolism	4	map00760
8	Purine metabolism	2	map00230
9	Fatty acid elongation	2	map00062
10	Arginine biosynthesis	4	map00220
11	Valine, leucine and isoleucine degradation	5	map00280
12	Benzoate degradation	2	map00362
13	Drug metabolism—cytochrome P450	2	map00982
14	Retinol metabolism	2	map00830
15	Biosynthesis of unsaturated fatty acids	5	map01040
16	Metabolism of xenobiotics by cytochrome P450	2	map00980
17	Phenylalanine metabolism	2	map00360
18	One carbon pool by folate	2	map00670
19	Nitrogen metabolism	4	map00910
20	Tyrosine metabolism	2	map00350
21	Pentose and glucuronate interconversions	3	map00040
22	Histidine metabolism	2	map00340
23	Butanoate metabolism	3	map00650
24	Chloroalkane and chloroalkene degradation	2	map00625
25	Glycerophospholipid metabolism	3	map00564
26	Folate biosynthesis	2	map00790
27	Carbon fixation pathways in prokaryotes	2	map00720
28	beta-Alanine metabolism	4	map00410
29	Glycine, serine and threonine metabolism	7	map00260
30	Fatty acid degradation	6	map00071
31	Carbon fixation in photosynthetic organisms	2	map00710
32	Drug metabolism—other enzymes	2	map00983
33	Ascorbate and aldarate metabolism	3	map00053
34	Alanine, aspartate and glutamate metabolism	4	map00250
35	Fatty acid biosynthesis	7	map00061
36	Arginine and proline metabolism	3	map00330
37	Propanoate metabolism	3	map00640

## Data Availability

The original contributions presented in this study are included in the article. Further inquiries can be directed to the corresponding author(s).

## Supplementary Materials

The following supporting information can be downloaded at https://www.mdpi.com/article/10.3390/ani15121807/s1. Table S1: Mapped DEGs by the RNA-Seq Analysis and Log2 Fold Change values; Table S2: KEGG Analysis.
